# Regulatory insights underlying apple to cold stress

**DOI:** 10.1093/hr/uhag080

**Published:** 2026-03-03

**Authors:** Yanfeng Jia, Jiarui Li, Mengyao Wei, Chaofan Li, Jiawang Qin, Mengjie An, Dongliang Guo, Quanlin Li

**Affiliations:** Xinjiang Key Laboratory of Biological Resources and Genetic Engineering, College of Life Science and Technology, Xinjiang University, Urumqi 830046, China; Xinjiang Key Laboratory of Biological Resources and Genetic Engineering, College of Life Science and Technology, Xinjiang University, Urumqi 830046, China; Xinjiang Key Laboratory of Biological Resources and Genetic Engineering, College of Life Science and Technology, Xinjiang University, Urumqi 830046, China; Xinjiang Key Laboratory of Biological Resources and Genetic Engineering, College of Life Science and Technology, Xinjiang University, Urumqi 830046, China; Xinjiang Key Laboratory of Biological Resources and Genetic Engineering, College of Life Science and Technology, Xinjiang University, Urumqi 830046, China; Xinjiang Key Laboratory of Biological Resources and Genetic Engineering, College of Life Science and Technology, Xinjiang University, Urumqi 830046, China; Xinjiang Key Laboratory of Biological Resources and Genetic Engineering, College of Life Science and Technology, Xinjiang University, Urumqi 830046, China; MOA Key Lab of Pest Monitoring and Green Management, College of Plant Protection, China Agricultural University, Beijing 100193, China

## Abstract

Apple (*Malus domestica*) is one of the most widely cultivated and consumed fruits worldwide, valued for its rich nutrition and health benefits. Low temperature (LT) significantly limits apple growth, productivity, and fruit quality. Understanding the intricate regulatory networks, underlying cold tolerance is crucial for developing resilient apple cultivars. In this review, we comprehensively summarize the molecular control enabling apple to withstand cold stress, encompassing transcription cascades, phytohormonal networks, reactive oxygen species (ROS) regulation, epigenetic modifications, and post-translational modifications (PTMs), as well as the crosstalk with drought, immune, and light signaling pathways. We also discuss the management strategies for enhancing apple cold tolerance, including the exploitation of wild germplasm resources, multi-omics-based network integration, gene editing, molecular marker-assisted breeding, rootstock grafting, and emerging bioinoculants approaches. This review provides a foundational framework for molecular breeding and gene regulatory strategies to improve cold resilience in apple.

## Introduction

Apple (*Malus domestica*), a major temperate perennial fruit crop, is one of the most widely consumed fruits globally. Its productivity is vulnerable to low temperature (LT), which causes developmental delays and fruit abscission [1]. In China, varieties ‘Hongjiangjun’, ‘Fushi’, ‘Hanfu’, ‘Qinguan’, and ‘Jinguan’ have been widely used to develop cold-resistant varieties. However, the molecular and genetic mechanisms underlying their cold resistance remain poorly understood.

Cold stress, comprising chilling (0°C–15°C) and freezing (<0°C) conditions, impairs plant growth, development, yield, quality, and geographical distribution [2]. To survive under cold stress, temperate plants adopt cold acclimation (CA) strategy to enhance environmental adaptability [3]. CA triggered by exposure to low nonfreezing temperatures initiates a set of regulatory hubs that remodel morphological changes, physiological processes, and biochemical progress. This process involves transcriptional control and epigenetic regulation of the inducer of CBF expression 1 (ICE1)-C-repeat binding factor (CBF)-cold-regulated gene (COR) (ICE1-CBF-COR) transcription cascade, reactive oxygen species (ROS) homeostasis and phytohormone signaling, and post-translational modifications in cold-signaling pathways [4]. Together, they enhance plants cold tolerance.

Recent progress has been made in elucidating the physiological and molecular basis of cold tolerance in apple [5]. Nevertheless, a comprehensive synthesis of these findings, particularly at the systems level, is still lacking. In this review, we summarize the latest progress in the molecular regulation of cold response in apple, covering transcriptional, epigenetic, and protein modification layers. We further investigate the crosstalk of these molecular regulatory events with other factors, including light, drought, and immunity. Our objective is to provide a comprehensive overview of cold tolerance regulation in apple and to propose prospective strategies for enhancing LT resilience.

## Cold-mediated transcriptional regulatory networks in apple

**Figure 1:**
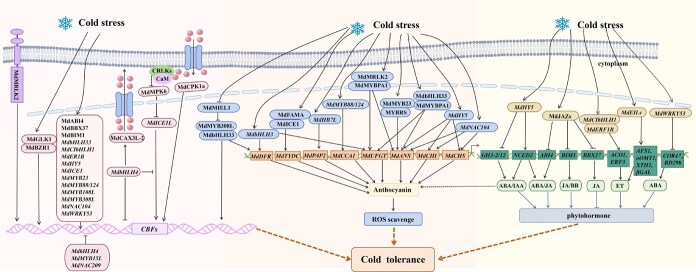
Cold stress triggers transcriptional regulatory networks in apple.

LT perception triggers extensive transcriptional reprogramming, leading to the coordinated activation of *COR* genes that orchestrate metabolic remodeling and stress-induced modulation of phytohormone signaling networks [5]. These integrated molecular responses collectively enhance plants cold tolerance (Fig. 1) (Table 1).

### The CBF-dependent transcriptional cascade and upstream modulation

CBF/Dehydration-responsive element‐binding protein (DREB) transcription factors (TFs) can directly bind the C-repeat/Dehydration-Responsive Element (CRT/DRE) elements in the promoters of *COR* genes to activate their transcription, termed *CBF*-dependent cold tolerance [6]. In apple, specific CBF members have been functionally validated: *MdCBF1* and *MdCBF2* directly activate the vacuolar sugar transporter genes *MdTST1* and *MdTST2* under LT conditions, promoting sugar accumulation as a compatible osmolyte [7]. *ICE1*, a basic helix–loop–helix (bHLH) TF, serves as the central regulator of *CBF* and *COR* genes [8]. Upon cold stress, ICE1 is post-translationally activated and directly bind to canonical MYC-recognition elements (MYC-REs) within the promoters of *CBF* genes, thereby activating *CBF* transcription and initiating downstream *COR* genes expression [7]. In apple, *MdICE1* expression is transcriptionally induced by LT, and the resulting MdICE1 protein sequentially up-regulates *MdCBFs*, establishing a feed-forward loop that amplifies the cold acclimation response [1, 9, 10–12].

Immediately upstream of the ICE1-CBF module, cytosolic Ca^2+^ acts as a universal second messenger that is rapidly mobilized across the plasma membrane within seconds of LT perception, providing the earliest detectable trigger for downstream transcriptional cascades. This cold-elicited Ca^2+^ flux activates calcium-dependent protein kinases, exemplified by MdCPK1a, which enhances antioxidant enzyme activity to scavenge ROS and directly phosphorylates ICE1/CBF proteins to potentiate *CBF* genes expression [13]. Additionally, plasma membrane-localized Ca^2+^/CaM-regulated receptor‐like kinase (CRLKs), which are regulated by Ca^2+^/calmodulin (CaM), can modulate the stability of the ICE1 protein via MdMPK6 [14]. To prevent cytotoxic Ca^2+^ overload, members of the cation/proton exchanger (CAX) family, including MdCAX3L-2, MdCAX2Ls, and MdCAX5Ls, actively sequester excess Ca^2+^ into the vacuoles, thereby compromising cold hardiness [10, 11]. Supporting this, MdCAX3L-2 alleviates *MdCBF1/3* transcriptional output, representing a feedback attenuation mechanism [14]. Thus, Ca^2+^-mediated activation coupled with CAX-dependent attenuation provides a biphasic regulatory switch that fine-tunes the robustness and duration of the ICE1-CBF-COR transcriptional cascade in apple.

Beyond the ICE1-CBF backbone, a multi-family transcriptional matrix refines the intensity and duration of CBF-dependent cold tolerance in apple. Positive regulators include members of at least nine TF families that physically associate with CBF promoters: (I) APETALA2/ethylene-responsive factor (AP2/ERF) proteins MdABI4, and MdERF1B intensify *MdCBF1* transcription under prolonged cold exposure [15, 16]; (II) bHLH factors *MdbHLH33* and *MdBIM1* boost *MdCBF1/2* expression [11, 17, 18]; (III) B-box (BBX) protein MdBBX37 [16] directly binds the promoters of both *MdCBF1* and *MdCBF4* to activate their transcription and physically interacts with MdICE1 to co-activate *MdCBF1* expression, thereby amplifying the CBF-dependent cold-response output in apple [19]; (IV) the basic Leucine Zipper (bZIP) *TF ELONGATED HYPOCOTYL 5* (*MdHY5*) positively regulates *MdCBF1* transcription through direct binding to the G-box motif in its promoter, thereby enhancing plants cold tolerance [20]; (V) MYB TFs *MdMYB23*, *MdMYB88/124*, *MdMYB108L*, and *MdMYB308L* function as either direct transcriptional activators or co-activators of *CBF* genes, thereby providing multi-layered positive control that reinforces the CBF-dependent cold-response network in apple [18, 21–23]; (VI) NAC representative *MdNAC104* physically interacts with the promoters of *MdCBF1* and *MdCBF3* to elevate their transcript abundance, activating the CBF-dependent cold-response pathway [24]; (VII) WRKY representative *MbWRKY53* contributes to plants tolerance to cold via the CBF pathway [25]; (VIII) *BRASSINAZOLE-RESISTANT1* (*MdBZR1*) can directly transcribe and activate *CBF1/2* [26]; and (IX) FERONIA receptor-like kinase gene *MdMRLK2* induces the expression of *MdCBF1/2/3* [27]. In contrast, to avoid the energetic overinvestment of sustained cold acclimation, apple deploys sequence-specific repressors that limit *CBFs* induction. The NAC-type repressor *MdNAC029* directly binds the *MdCBF1* and *MdCBF3* promoters, acting as a transcriptional brake that silences *CBFs* expression [28]. Concurrently, the R2R3-MYB repressor *MdMYB15L* attenuates the *MdCBF2*-mediated cold response through targeted transcriptional inhibition [29].

**Table 1:** Key factors involved in apple cold stress response

Pathway	Gene symbol	Functional characteristics	Cold treatment	Tissue	Effect	Target(s)/Directly	References
CBF- dependent							
	*MdABI4*	AP2/ERF TF	4°C/10 d; 4°C/1 d; 4°C/5 d	Calli; Leaves; Seedlings	Positive	*MdCBF1*	[15]
	*MdBBX37*	BBX family protein	4°C/10 d; 4°C/1 d; 4°C/5 d	Calli; Leaves; Seedlings	Positive	*MdCBF1/4*	[19]
	*MdbHLH4*	bHLH TF	4°C/3 h; −6°C/3 h, −8°C/4 h	Calli; Plants	Negative	*MdCBF1/3*	[14]
	*MdbHLH33*	bHLH TF	4°C/10 d, 14 d	Calli	Positive	*MdCBF2*	[11, 18]
	*MdBIM1*	bHLH TF	4°C/10 d; 4°C/1 d; 4°C/5 d	Calli; Leaves; Seedlings	Positive	*MdCBF1*	[17]
	*MdBZR1*	BZR1 TF	4°C/10 d; 4°C/1 d; 4°C/6 d	Calli; Leaves; Seedlings	Positive	*MdCBF1/2*	[26]
	*MdCAX3L-2*	CAX family	4°C/3 h; 4°C/12 h	Calli; Plants	Negative	*MdCBF1/3*	[14]
	*MdCBF1/2*	AP2/ERF TF	4°C/2 d, 4 d; 4°C/3 h, 6 h	Fruits; Plantlets	Positive	*MdTST1/2*	[7]
	*MdCIbHLH1*	bHLH TF	15°C/23 d; 0°C/7 d	Calli; Seedlings	Positive	*MdCBF2*	[1]
	*MdCPK1a*	CDPK family protein	4°C/10 d; 4°C/10 d	Plants; Seedlings	Positive	*MdCBFs*	[13]
	*MdERF1B*	AP2/ERFTF	4°C/7 d; 0°C/7 d	Calli; Seedlings	Positive	*MdCBF1*	[16]
	*MdGLK1*	GLK1 TF	4°C/10 d; 4°C/1 d; 4°C/6 d	Calli; Leaves; Seedlings	Positive	*MdCBF1/2*	[26]
	*MdHB7L*	HD-Zip TF	4°C/20 d; 4°C/35 d, 4°C/24 h; 4°C/12 h, −6°C/4 h, −8°C/4 h, −10°C/2 h	Calli; Leaves; Plants	Positive	*MdCBFs*	[46]
	*MdHY5*	bZIP TF	4°C/10 d, 14d	Calli	Positive	*MdCBF1*	[20, 23]
	*MdICE1*	bHLH TF	4°C/10 d; 4°C/10 h, 24 h; 4°C/12 h, −6°C/3 h; 4°C/5 d, 4°C/30 d	Calli; Leaves; Plantlets; Seedlings	Positive	*MdCBFs*	[12, 101]
	*MdICE1L*	bHLH TF	4°C/3 h; 4°C/12 h	Calli; Plants	Positive	*MdCBF1/3*	[46]
	*MdMPK6*	MAPK family protein	4°C/3 h; 4°C/12 h	Calli; Plants	Negative	*MdCBF1/3*	[14]
	*MdMRLK2*	FER family	12°C/15 d; −2°C, −4°C, −6°C/1 h; 4°C/30 d, −10°C/25 min; 4°C/30 d; −30°C, −35°C, −40°C/12 h	Calli; Leaves; Plants; Seedlings; Twigs	Positive	*MdCBFs*	[27]
	*MdMYB15L*	MYB TF	4°C/14 d	Calli	Negative	*MdCBF2*	[29]
	*MdMYB23*	R2R3-MYB TF	4°C/10 d	Calli	Positive	*MdCBF1/2*	[21]
	*MdMYB88/124*	MYB TF	4°C/30 d, 4°C/7 d, −10°C/20 min, 25 min, 30 min	Plants	Positive	*MdCBF3*	[22]
	*MdMYB108L*	MYB TF	4°C/14 d	Calli	Positive	*MdCBFs*	[23]
	*MdMYB308L*	MYB TF	4°C/10 d	Calli	Positive	*MdCBF2*	[18]
	*MdNAC029*	NAC TF	4°C/10 d	Calli	Negative	*MdCBF1/4*	[28]
	*MdNAC104*	NAC TF	4°C/20 d; 4°C/8 h; −5°C/5 h, −7°C/6 h	Calli; Leaves; Plants	Positive	*MdCBF1/3*	[24]
	*MbWRKY53*	WRKY TF	TF4°C/0 h, 2 h, 4 h, 6 h, 8 h, 12 h	Leaves; Roots	Positive	*MdCBF2A*	[25]
Anthocyanin/ROS							
	*MdbHLH3*	bHLH TF	17°C/0 min, 5 min, 15 min; 17°C/3 d	Calli; Plantlets	Positive	*MdUFGT*, *MdDFR*	[49]
	*MdbHLH33*	bHLH TF	4°C/10 d	Calli	Positive	*MdDFR*	[18]
	*MdHB7L*	HD-Zip TF	4°C/20 d; 4°C/35 d, 4°C/24 h; 4°C/12 h, −6°C/4 h, −8°C/4 h, −10°C/2 h	Calli; Leaves; Plants	Positive	*MdPAP2*	[46]
	*MdHY5*	bZIP TF	−10°C/25 min, 4°C/25 d	Plants	Positive	*MdANS*, *MdCHI*, *MdCHS*	[35]
	*MdMYB23*	MYB TF	4°C/10 d	Calli	Positive	*MdANR*	[21]
	*MdMYB88/124*	MYB TF	4°C/30 d, 4°C/7 d, −10°C/20 min, 25 min, 30 min	Plants	Positive	*MdCCA1*	[22]
	*MdMYB308L*	MYB TF	4°C/10 d	Calli	Positive	*MdDFR*	[18]
	*MdMYBPA1*	MYB TF	12°C, −2°C, −4°C, −6°C/1 h; 4°C/30 d, −10°C/25 min; 12°C/15 d; 4°C/30 d; −30°C, −35°C, −40°C/12 h	Calli; Leaves; Plants; Seedlings; Twigs	Positive	*MdANS*, *MdUFGT*	[27, 48]
	*MdNAC104*	NAC TF	4°C/20 d; 4°C/8 h; −5°C/5 h, −7°C/6 h	Calli; Leaves; Plants	Positive	*MdCHI*, *MdF3H*, *MdANS*, *MdCHS*	[24]
Hormone							
ET	*MdEILs*	EIL	3°C, 5°C/70 d	Fruits	Positive	*MdAFS1*, *MdXTH1*, *MdβGAL*	[40]
ET	*MdERF1B*	AP2/ERF TF	0°C/7d; 4°C/7 d	Seedlings; Calli	Positive	*MdACS1*, *MdACO1*, *MdERF3*	[16]
JA	*MdJAZs*	Jasmonate ZIM-domain protein	4°C/10 d; 4°C/1 d; 4°C/5 d	Calli; Leaves; Seedlings	Positive	/	[19]
ABA	*MbWRKY53*	WRKY TF	4°C/0 h, 2 h, 4 h, 6 h, 8 h, 12 h	Leaves; Roots	Positive	*COR47*, *RD29b*	[25]
ABA/JA	*MdABI4*	AP2/ERF TF	4°C/10 d; 4°C/1 d; 4°C/5 d	Calli; Leaves; Seedlings	Positive	/	[15]
JA/BR	*MdBIM1*	bHLH TF	4°C/10 d; 4°C/1 d; 4°C/5 d	Calli; Leaves; Seedlings	Positive	*MdCBF1*, *MdCORs*	[17]
IAA/ABA	*MdHY5*	bZIP TF	−10°C/25 min; 4°C/25 d	Plants	Positive	*MdGH3-2/12*, *MdNCED2*	[35]

### Phytohormonal circuits-mediated CBF-dependent and CBF-independent cold tuning

Plants coordinate developmental programs and stress responses through dynamic phytohormones networks. These chemical signals act as either positive or negative modulators of cold tolerance via CBF-dependent and/or independent mechanisms [30]. In this section, we summarize recent advances regarding the roles of abscisic acid (ABA), brassinosteroids (BR), ethylene (ET), jasmonic acid (JA), and indole-3-acetic acid (IAA), in regulating cold stress of apple.

ABA is pivotal endogenous phytohormone that coordinates plant growth, development, and abiotic stress adaptation. Following cold treatment, the application of exogenous ABA typically enhances cold tolerance. Cold stress stimulates endogenous ABA accumulation by inducing the expression of the ABA biosynthesis-related gene *NCED* and repressing the expression of the ABA catabolic enzyme-encoding gene *CYP707A* [31]. This cold-triggered ABA accumulation is functionally significant, which minimizes cold injury by stabilizing cell membranes and boosting antioxidant enzyme activities [32–34]. The ABA signaling component MdABI4 interacts with MdICE1 and enhances its transcriptional activation of the downstream target gene *MdCBF1*, thus improving ABA-mediated cold tolerance. This ABI4-ICE1-CBF module represents a critical hub linking ABA signaling to the core cold response pathway in apple. ABA-mediated cold tolerance is regulated through intricate crosstalk with other phytohormones. The JA-ZIM domain (JAZ) proteins MdJAZ1 and MdJAZ2 interfere with the interaction between MdABI4 and MdICE1 by interacting with MdABI4, thus integrating JA signal into ABA-dependent cold tolerance [15]. Similarly, the light-regulated TF *MdHY5* serves as a crucial hub integrating ABA and IAA pathways, which positively regulates cold tolerance by modulating the expression of auxin–amido synthetase-encoding genes *MdGH3-2/12* and ABA biosynthesis gene *MdNCED2*, thereby promoting the anthocyanins accumulation [35]. This dual regulation links ABA, IAA, and flavonoid metabolism, highlighting the metabolic reconfiguration that accompanies hormonal signal integration in apple cold adaptation. Unlike *MdHY5*, *MbWRKY53* enhances plants tolerance to cold stress by upregulating the transcription of stress-related genes involved in the CBF pathway, salt overly sensitive (SOS) pathway, proline synthesis , and ABA signaling components [25].

BR are plant-specific steroid hormones that rapidly re-programme transcriptional and translational landscapes under LT. Exogenous 24-epibrassinolide significantly elevates transcript levels of *CBF1* in *Arabidopsis* [33, 36]. Studies on BR signal-defective mutants showed that BR augment plants cold tolerance by modulating *CBFs* transcriptional cascades through both translational control and post-translational modification [37, 38]. Instead, BES1-INTERACTING MYC-LIKE1 (MdBIM1) not only physically binds the promoter of *MdCBF1* to activate its transcription, while simultaneously forming a transcriptional complex with *MdCBF2* to potentiate *MdCBF2*-activated expression of downstream *COR* genes, but also merges BR and JA signaling by nucleating a JAZ-BIM1-CBF module [17].

ET plays dual roles in the cold stress response of various plants [39]. In apple, freezing tolerance is associated with increased ET levels. Cold-induced ET release is associated with *MdERF1B*, which positively regulates cold tolerance and ET biosynthesis in an MdCIbHLH1-dependent manner [16]. The TF *MdEIL1-4* silenced lines exhibited reduced softening after cold treatment and decreased induction of cold-related genes [40].

JA is a lipid-derived phytohormone whose endogenous contents rise by cold-triggered JA biosynthesis. Applying exogenous JA can boost plants cold tolerance by increasing *CBFs* expression, whereas genetic blockade of JA biosynthesis and signaling confers freezing hypersensitivity [41]. In *Arabidopsis*, under normal temperatures, the JA signaling repressors JAZ1 and JAZ4 physically interact with ICE1/2 to inhibit their transcriptional activity. However, under cold stress conditions, the JA receptor coronatine insensitive 1 (COI1) degrades JAZ proteins to release ICE1, which activates *CBF* genes expression and enhances cold tolerance [42]. The apple orthologs MdJAZ1/2 execute an analogous role in regulating JA-mediated cold tolerance through interfering with three documented activator modules [15, 17, 19]. First, MdJAZ1/2 block the MdABI4-MdICE1 interaction, preventing MdABI4 from potentiating ICE1-dependent *CBF* expression and consequently lowering ABA-dependent freezing resistance [15]. Second, MdJAZ1/2 physically associate with MdBIM1 to repress its activation of *MdCBF1* expression and interfere with MdBIM1-MdCBF2 complex formation, thereby alleviating *MdBIM1*/BR-promoted cold stress tolerance [17]. Third, MdJAZ1/2 physically interact with MdBBX37 to prevent the activation of the core CBF-dependent cold response pathway [19]. By this triple-target strategy, MdJAZ1/2 integrate JA status into ABA, BR, and light signaling networks, providing a tunable checkpoint that calibrates apple cold acclimation.

 Accumulating evidence positions phytohormones as pivotal modulators of apple cold tolerance, whereas the molecular mechanisms governing regulatory roles remains largely uncharacterized. Future efforts should therefore focus on elucidating the precise functions and molecular pathways through which phytohormones mediate cold stress responses. Moreover, the contributions and mechanistic basis of crosstalk among cytokinins (CK), strigolactones (SL), and gibberellic acid (GA) in apple cold resistance merit further investigation and should be incorporated into the hormone network-driven cold adaptation framework.

### ROS-centric regulatory networks in cold hardiness

Upon LT perception, plant cells rapidly generate the secondary messengers Ca^2+^, ROS, nitric oxide (NO), and 2’,3’-cAMP to propagate the cold signal [43]. Cold-gated Ca^2+^ channels elevate cytosolic Ca^2+^ levels, thereby activating CIPKs, CaM/CML, CAMTA, and CDPK modules that feed into both CBF-dependent and CBF-independent transcriptional programmes. ROS, NO, and 2’,3’-cAMP cooperate with Ca^2+^ to fine-tune this response. In apple, empirical evidence for secondary messenger-mediated refinement of cold signal transduction remains scarce. Conversely, ROS exhibits concentration-dependent functions and are well established as cytotoxic when overaccumulated.

LT triggers the overproduction of ROS, which disrupt the electron transport chain and are toxic to cellular processes [44]. To mitigate ROS-caused damage, plants utilize both enzymatic and non-enzymatic antioxidants to eliminate excess ROS. The main enzymatic antioxidants include peroxidase (POD), superoxide dismutase (SOD), catalase (CAT), glutathione peroxidase (GPX), ascorbate peroxidase (APX), and glutathione S-Transferase (GST) [45], which are generally induced by cold stress and play a crucial role in ROS scavenging. The non-enzymatic antioxidants like L-ascorbic acid (AsA), Glutathione (GSH), and anthocyanin provide additional reducing power to neutralize excess ROS. During cold-induced ROS scavenging process, TFs of the bZIP, MYB, TCP, and NAC families bind to the promoters of antioxidant enzyme promoters, thereby enhancing cold tolerance [32]. The HD-Zip protein, MdHB7L upregulates *MdPRX52/53* and the dehydration-responsive gene *MdRD26* to enhance apple antioxidative capacity under chilling stress [46].

Anthocyanins are an important cold-induced non-enzymatic antioxidants [32], whose biosynthesis is controlled by enzymes and regulatory factors [47]. In apple, MdMYB23 promotes proanthocyanidin accumulation and ROS scavenging by activating *MdANR* transcription [21]. Similarly, *MdMYB88* and *MdMYB124* target *MdCSP3* and *MdCCA1*, respectively, to increase anthocyanin-mediated cold protection [22]. Likewise, the MdMYBPA1–MdbHLH33 complex activates *MdANS* and *MdUFGT* to accelerate anthocyanin synthesis and ROS removal [27, 48]. Moreover, MdbHLH3 interacts with MdMYB1 to promote the expression of *MdUFGT* and *MdDFR*, thereby activating anthocyanin biosynthesis and enhancing fruit coloration [49]. Multiple TFs can simultaneously initiate anthocyanin- and antioxidant enzyme-dependent cold tolerance. For instance, *MdNAC104* targetedly upregulated *MdCHS-b*, *MdCHI-a*, *MdF3H-a*, and *MdANS-b* transcription to promote anthocyanin synthesis, as well as *MdFSD2*, and *MdPRXR1.1* to increase enzyme activity [24]. Another example is *MdHY5*, which upregulates the expression of *MdANS*, *MdCHI*, and *MdCHS* and enhance POD-, SOD-, and CAT-mediated ROS clearing in response to LT stress [35]. Beyond the flavonoid-antioxidant axis, alternative metabolic modules are being elucidated. The MdICE1/MdFAMA-*MdTYDC* regulatory module confers cold tolerance by orchestrating dopamine metabolism [12].

 The emerging portrait establishes a ROS-centric detoxification network that integrates CBF-independent transcriptional reprogramming with metabolite-based antioxidant systems to secure apple cold hardiness. Future research should identify novel ROS-detoxification regulatory modules and clarify how ROS, acting as a second messenger, allocates resources between CBF and non-CBF branches under fluctuating field temperatures. Particularly, the molecular basis by which ROS coordinates cold-signal transmission with Ca^2+^ dynamics at the plasma-membrane-cytosol interface warrants investigation.

## Epigenetic regulation of apple cold adaptation

**Figure 2:**
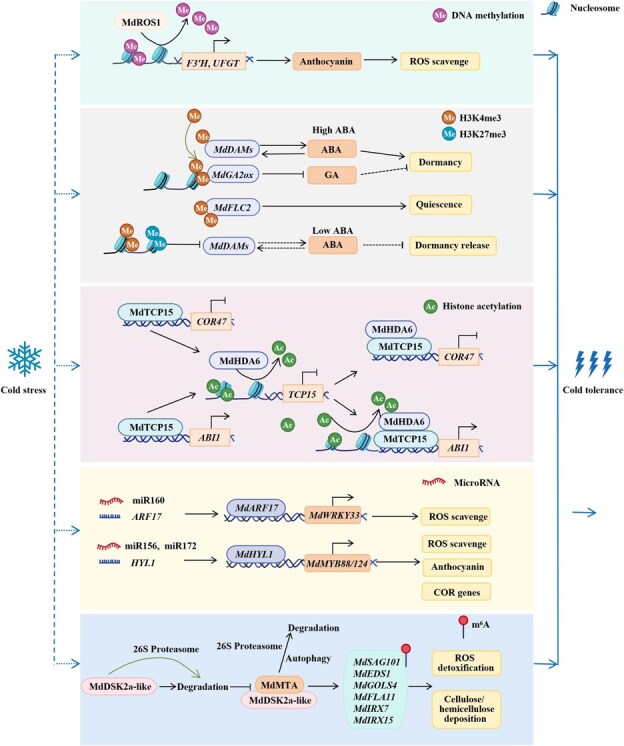
Cold stress regulates gene expression through epigenetic modulation, including DNA methylation, histone methylation, histone acetylation, microRNAs (miRNAs) and m^6^A, play critical roles in regulating cold tolerance-associated genes.

Beyond transcriptional networks, epigenetic mechanisms operate as central players in plants cold-stress responses [50]. DNA methylation, histone modifications, chromatin remodeling, RNA modifications collectively gate the accessibility of cold-mediated loci, ensuring rapid yet reversible induction of freezing tolerance; whereas chemical modifications and noncoding RNAs (ncRNAs) jointly mediate the stability of cold-inducible mRNA at post-transcriptional levels, ensuring controllable cold response [51]. Recent studies have further illuminated how each layer modulates the apple CBF-dependent and CBF-independent cold tolerance pathways (Fig. 2).

### DNA methylation

DNA methylation is a fundamental and conserved epigenetic mark that regulates plant development and stress responses by maintaining genome integrity and controlling gene expression [52]. In plants, DNA methylation occurs in various sequence contexts, including CG, CHG, and CHH, where H represents A, T, or C. Furthermore, DNA methylation levels are dynamically regulated by both methylation and demethylation processes [51]. Currently, the investigation on DNA methylation in apple mainly focuses on development [53], drought resistance [54, 55], anthocyanin biosynthesis [56], and its role in LT stress is now gaining attention [57, 58]. Compared with high chill conditions, under low chill conditions, demethylation levels decrease from the dormant bud to the fruiting stage in apple, while methylation levels increase [57]. Yu *et al.* demonstrated *that the DNA demethylase MdROS1* promotes LT-induced anthocyanin accumulation by reducing methylation levels in the promoters of the anthocyanin biosynthesis genes *MdF3H* and *MdUFGT* [58].

### Histone modifications

#### H3K4me3 and H3K27me3

Histone H3 lysine 4 trimethylation (H3K4me3) universally marks active transcription [59], whereas euchromatin-enriched H3K27 trimethylation (H3K27me3) represents a repressive mark and exhibits tissue specificity [60]. Under LT conditions, H3K27me3 and H3K4me3 function as memory marks for the transcriptional activity of *COR* genes in *Arabidopsis*, potato, and cucumber, respectively [61–63]. In apple, prolonged chilling induces the expression of the MADS-box TF *FLOWERING LOCUS C* (*FLC*)-like gene, which positively correlates with changes in H3K4me3 marks, whereas lower expression under warmer conditions negatively correlates with the H3K27me3 levels [64]. Conversely, prolonged LT increases H3K27me3 levels at the *DORMANCY ASSOCIATED MADS-box* (*DAM*) locus to inhibit its expression [65]. This repressive signature is complemented by reduced H3K4me3 on gibberellin metabolism genes, collectively orchestrating the temporal control of the chilling-induced dormancy cycles [66].

### Histone acetylation

Histone acetylation is one of the most extensively studied histone modifications and is controlled by histone acetyltransferases (HATs)-mediated acetylation and histone deacetylases (HDACs)-dependent deacetylation, mirroring the gene activation and repression, respectively [67–69]. Although the HAT-HDAC circuitry that underpins cold acclimation has been dissected in *Arabidopsis*, rice, and maize [70–74], its function in apple is only now emerging. Jiang *et al.* identified 58 *MdHATs* from the apple genome and showed that tobacco overexpression *MdHAG1* exhibits reduced cold tolerance, whereas *MdHAM1* -overexpressing lines display enhanced tolerance, underscoring isoform-specific roles [75]. On the deacetylation side, Guo et al. demonstrated that MdHDA6 deacetylates histones within the Teosinte Branched 1, Cycloidea and Proliferating Cell Factors 15 (MdTCP15) promoter to silence its transcription and physically associates with MdTCP15 to quench its activation capacity. This dual repression locks downstream *COR* genes in an off state, ultimately enhancing apple cold resistance [67].

### m6A 

*N* ^6^-Methyladenosine (m^6^A) is the most prevalent internal chemical modification in eukaryotic mRNA, modulating gene expression during both transcriptional and post-transcriptional processes [76]. This modification is dynamically maintained by methyltransferases (writers), reader proteins (readers), and demethylases (erasers) [77, 78]. In plants, the multi-protein complex responsible for catalyzing m6A modifications includes methyltransferase A (MTA), methyltransferase B (MTB), FKBP12 INTERACTING PROTEIN 37 KD (FIP37), VIRILIZER (VIR), HAKAI, and HAKAI-interacting zinc finger protein (HIZ2) [79]. In apple, *MdMTA* deposits m^6^A on *COR* transcripts under cold stress, enhancing their stability and promoting ROS detoxification alongside cellulose and hemicellulose deposition, thereby enhancing cold tolerance. Conversely, MdDSK2a-like, a ubiquitin receptor protein, reduces the m^6^A levels on *MdMTA* target genes through degrading MdMTA via the 26S ubiquitin-dependent proteasome and autophagy pathways. This deposition inhibits ROS scavenging and weakens cell-wall reinforcement, thus compromising cold tolerance [80].

### Noncoding RNAs 

Several recent reviews have discussed the current advancements in ncRNAs involved in plants cold response [81, 82]. Small non-coding RNAs (sncRNAs), including microRNAs (miRNAs) and small interfering RNAs (siRNAs), have also been isolated and characterized in apple [83–89]. However, their functional characterization remains predominantly skewed toward development, drought, salt stress, immune response, and anthocyanidin biosynthesis [83–85, 90–93]. Till now, only two reports have investigated cold-resistant Mdm-miRNAs in apple. Mdm-miR160 negatively regulates cold stress by targeting *MdARF17*, which in turn targetedly activates *MdWRKY33* expression. The Mdm-miR160-*MdARF17-MdWRKY33* regulatory module impairs ROS scavenging-mediated cold tolerance by accumulating H2O2 and decreasing POD and CAT enzyme content [94]. Additionally, the RNA-binding protein HYPONASTIC LEAVES1 (MdHYL1) regulates the biogenesis of several cold-related Mdm-miRNAs, including the negative regulator Mdm-miR156 and the positive regulator Mdm-miR172 [95]. Beyond these examples, LT-inducible siRNAs, long non-coding RNAs (lncRNAs) and circular RNAs (circRNAs) remain completely unexplored in apple, representing a conspicuous knowledge gap for future epigenomic surveys. 

 Overall, apple cold epigenomics remains in its infancy. Future priorities should integrate multi-omic datasets (methylome, acetylome, m^6^A-ome, ncRNA-ome) with high-resolution spatio-temporal transcriptomes to build a systems-level epigenetic map that reveals how apple perceives, remembers and transmits cold signals across growing seasons.

## Post-translational modifications of apple cold signalling and response

**Figure 3:**
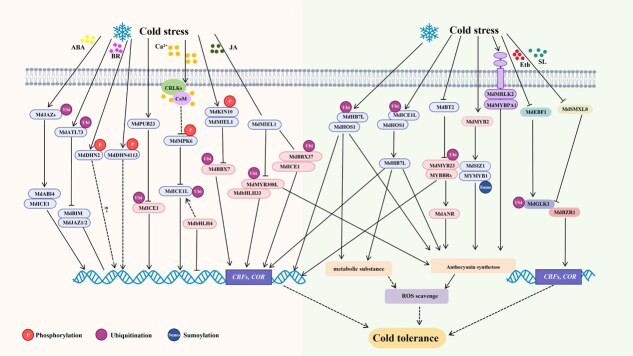
Post-translational modifications switch protein activity in response to cold stress.

Cold sensing in apple extends beyond mere transcriptional regulatory networks, which are rapidly converted into chemical tags on proteins. Post-translational modifications (PTMs), including phosphorylation/dephosphorylation, ubiquitination, SUMOylation, and glycosylation, rewire signaling cascades and adjust protein half-life and function, redirecting metabolic fluxes that ultimately determine freezing survival [5, 96]. In this section, we outline the post-translational regulatory processes that underpin cold resistance in apple (Fig. 3).

### Phosphorylation

Phosphorylation is the principal PTM for amplifying cold signals and refining cold responses through kinase-mediated activation of receptors, enzymes, and TFs. In *Arabidopsis*, CRLK1/2 restrain cold-induced MPK3/6 to stabilise ICE1, and boost *CBFs* expression [97], whereas CRPK1 phosphorylates 14-3-3 proteins to escort *CBF1/3* to ubiquitin-mediated degradation [98]. In parallel, the MEKK1-MAPKK2-MPK4 cascade positively regulates cold tolerance by constitutively inhibiting MAPK3/6 activity [99]. By contrast, phosphorylation-regulated cold tolerance in apple has only two reported cases. One is the FERONIA receptor-like kinase MdMRLK2, which associates with MdMYBPA1 to enhance its binding to the *MdANS* and *MdUFGT* promoters, thereby enhancing more anthocyanin-dependent freezing tolerance [48]. The other is the cold-inducible phosphorylation of dehydrins MdDHN2 and MdDHN4 [100].

### Ubiquitination

Ubiquitination is another pivotal PTM in response to environmental stimuli, which enables plants to precisely modulate protein turnover of stress-triggered signal transducers and stress-responsive regulators. Cold stress directly suppresses components of the ubiquitination machinery, thereby stabilizing key positive regulators of cold acclimation. LT reduces the abundance of the adaptor protein MdBT2 , thereby blocking MdBT2-mediated degradation of the MdMYB23 protein. This stabilization of MdMYB23 promotes proanthocyanidin-dependent freezing tolerance [21]. The E3 ubiquitin ligase MdPUB23 ubiquitinates the key cold signaling integrator MdICE1 and promotes its degradation via the 26S proteasome pathway, attenuating MdICE1--dependent *CBF* output [15, 101]. MYB30-INTERACTING E3 LIGASE 1 (MdMIEL1) mediates the ubiquitination and degradation of MdMYB308L and MdBBX37 to reduce MdMYB308L-activated anthocyanin accumulation and MdBBX37-controlled transcriptional cascade, respectively [18, 19]. Notably, the energy sensor MdKIN10 phosphorylates MdMIEL1 to trigger its autophagic degradation, releasing *MdBBX7* to activate *MdFAD8* and *MdCBF2* transcription. *MdFAD8* promotes fatty acid desaturation to maintain membrane fluidity, while *MdCBF2* drives *COR* genes expression, thereby coordinately enhancing apple cold tolerance [102]. Additional layers are provided by ARABIDOPSIS TOXICOS en LEVADURA73 (MdATL73), which ubiquitinates transcriptional activator MdBIM1 to dampen CBF-dependent resistance pathways [17]. During the early stages of cold stress, E3 ubiquitin ligase MdHOS1 interacts with MdICE1L and mediates its ubiquitination and proteasomal degradation, thereby promoting the accumulation of MdHB7L protein. Subsequently, MdHB7L enhances plants cold tolerance through both CBF-dependent and CBF-independent pathways. Upon prolonged cold exposure, depletion of MdICE1L causes MdHOS1 to target and degrade MdHB7L, repressing its cold-mediated signaling [46]. MdEBF1, a repressor in the ET signaling pathway, mediates the ubiquitination and degradation of MdGLK1. MdGLK1 enhances apple tolerance to cold stress by facilitating *MdBZR1*-mediated transcriptional activation of *MdCBF1/2*. Conversely, exogenous application of strigolactone (SL) represses MdSMXL8, which disrupts the interaction between MdGLK1 and MdBZR1, thereby impairing the MdGLK1-mediated regulatory mechanism of cold tolerance [26].

### SUMOylation

Small ubiquitin-like modifier mediated modification (SUMOylation) represents a crucial post-translational regulatory mechanism for rapid signal transduction and transcriptional reprogramming in plant stress responses. SUMO E3 ligase MdSIZ1 sumoylates and stabilizes the MdMYB1 protein to promote anthocyanin biosynthesis in response to cold stress [103].

 In apple-cold interactions, ubiquitination dominates the current PTM landscape, whereas phosphorylation and SUMOylation are sparsely documented. Other PTM types, such as dephosphorylation and glycosylation, represent critical knowledge gaps. Advancing research in these underexplored areas will illuminate how apple coordinates multi-layered PTM codes to survive winter.

## The interplay between cold-stress responses and other factors

 Environmental signals rarely operate in isolation, and emerging evidence reveals extensive crosstalk among cold responses and the light, drought, and immune signaling networks. Understanding these interconnected pathways is crucial for deciphering how apple coordinates complex adaptive strategies under field conditions where multiple stresses converge.

### Crosstalk between light and cold signaling

**Figure 4:**
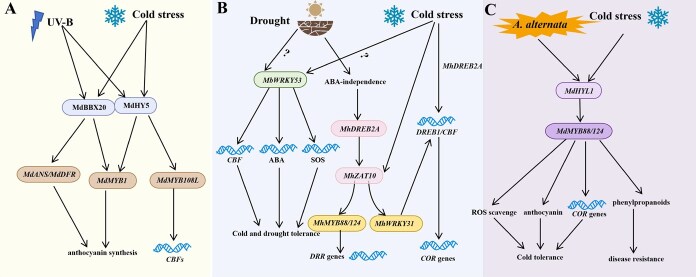
Crosstalk among cold signaling, light, drought, and immune responses in apple.

Light and temperature are two pivotal environmental cues that coordinate plant growth and development, with their integration emerging as a key research focus in plants stress physiology [104]. LT directly impairs photosynthetic electron transfer efficiency [105], while the core cold-mediated *CBF* regulation is itself modulated by light quality, photoperiod, and circadian rhythm, all of which are highly dependent on the light environment [106, 107]. Beyond CBF, *HY5* acts as the master regulator of light signaling to control the expression of the downstream genes, thereby regulating physiological and developmental processes in plants [104]. *HY5* also plays critical roles in controlling the responsiveness of plants to LT, involving the gene expression regulation, hormone homeostasis, and antioxidant synthesis. In gene expression, *MdHY5* modulates the expression of cold-induced genes in apple through CBF-dependent and CBF-independent pathways [20]. *MdHY5* also upregulates *MdMYB108L*-mediated *MdCBF3* expression [23]. As for hormone homeostasis, MdHY5 targets and inhibits *MdGH3*-*2/12* expression to reduce IAA content, while also activating *MdNCED2*-mediated ABA biosynthesis [35]. Regarding anthocyanin accumulation, MdHYH5 interacts with UV-B and cold-induced MdBBX20 to synergistically increase the expression of *MdMYB1*, *MdANS*, and *MdDFR*, thereby promoting anthocyanin synthesis [108] (Fig. 4).

### Crosstalk between drought and cold pathway

Drought and cold are the most pervasive abiotic stresses constraining plant growth, development, and geographical distribution. Emerging evidence demonstrates that plants pretreated with drought stress can establish systemic stress memory, significantly improving their tolerance to LT through coordinated reprogramming of multiple physiological and molecular pathways, including hormone signaling, ROS homeostasis, osmotic adjustment, energy metabolism, and secondary metabolism [109, 110]. In apple, transcriptomic analyses have revealed that most drought-responsive genes are also induced by cold, suggesting substantial overlap in downstream regulatory events and a common transcriptional framework for stress adaptation [111]. Central to this crosstalk is a hierarchical TF module comprising *MhDREB2A*, which directly regulates the zinc finger TF *MhZAT10*. *MhZAT10*, in turn, enhances stress tolerance by activating downstream targets *MhWRKY31* and *MhMYB88/124* [112]. Likewise, the WRKY TF *MbWRKY53* contributes to both cold and drought tolerance through the CBF pathway, SOS pathway, proline synthesis pathway, and ABA-dependent pathway [25] (Fig. 4).

### Crosstalk between immune response and cold pathway

Plants deploy synergistic or antagonistic strategies to combat concurrent biotic and abiotic stresses through extensive regulatory crosstalk units. SA is a crucial central regulatory molecule that links plants immunity and cold resistance. In *Arabidopsis*, ICE1 directly activates the SA signaling pathway, thereby enhancing antipathogen immune response under cold stress [113–115]. Apple leaf spot, caused by *Alternaria alternata* (*A. alternata*), is one of the most devastating diseases in the apple industry. HYL1 contributes to freezing tolerance and resistance to *A. alternata* by modulating the transcription of *MdMYB88* and *MdMYB124* [22, 95]. These MYB-type TFs enhance cold hardiness by inducing both CBF-independent *MdCSP3* transcription and CBF-dependent *MdCCA1* expression [22], while their downstream target *MdCM2* promotes phenylpropanoids accumulation against *A. alternata* infection. *MdHYL1* also regulates the biogenesis of Mdm-miR156, Mdm-miR172, and Mdm-miR160, conferring dual tolerance to cold and *A. alternata* [116] (Fig. 4).

 The crosstalk between LT and other environmental factors has been preliminarily charted in apple, while the key nodes that simultaneously integrate these cues remain poorly characterized and the corresponding synergistic or antagonistic networks have not been systematically dissected. Therefore, there is an urgent need to combine genetic screening with multi-omics approaches, including transcriptomics, epigenomics, proteomics and metabolomics to identify and functionally validate core regulatory genes that merge diverse environmental inputs into a coherent cold-response output.

## Future outlook and strategy

**Figure 5:**
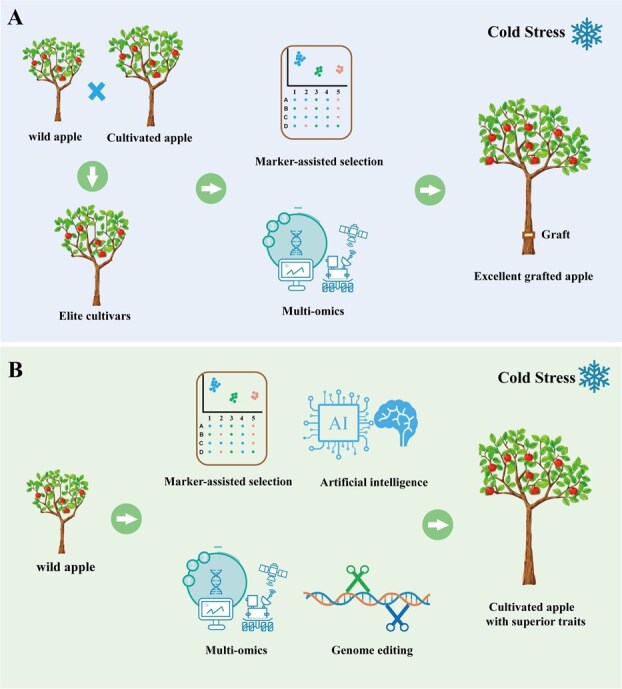
Integrated rootstock grafting and gene editing strategies for cold-resistant apple breeding.

LT stress significantly affects the growth, yield, and fruit quality of apples, making it a major limiting factor in apple production. Breaking this bottleneck demands a coordinated pipeline that couples germplasm mining, artificial intelligence (AI)-driven multi-omics, precision breeding, gene editing, rootstock grafting, and bioinoculants. Deep integration of multi-omics is the keystone, enabling structured collection, comprehensive identification, and gene mining of apple germplasm resources. This foundation facilitates the development of efficient data-sharing platforms, and ultimately delivers targeted improvement of apple varieties (Fig. 5).

### Wild germplasm mining and allele discovery

Apple wild germplasm resources harbor abundant cold-adapted alleles and structural variants, and wild *Malus species* represent an underexploited source of resilient rootstock. A pan-genome map generated from 29 wild apple species and one cultivated accession reveals that domestication has eroded cold and disease resistance via loss-of-function mutations in *MdMYB5* [117]. Notably, *Malus baccata* (*M. baccata*) achieves dual tolerance to LT and drought through the *MbbHLH93*-CBF pathway and coordinated accumulation of soluble sugars and antioxidant enzyme activity [118]. Future work should combine deep resequencing of altitudinal and latitudinal ecotypes with high-throughput phenomics under controlled freezing conditions to uncover novel alleles for downstream validation and rootstock selection.

### High-resolution QTLs and Molecular markers

Molecular marker bridge wild reservoirs and elite cultivars. Quantitative trait locis (QTLs) mapping and Genome-Wide Association Study (GWAS)convert cold-resistant genetic loci in F1 apple populations into high-throughput markers. Dominant apple cold-resistant QTLs have been identified on chromosomes LG08 and LG09. Notably, the QTL interval on LG09 contains the cell cycle regulatory gene *MdERF1B*, *MdPRPs*, and *MdPRX10*, which enhance cold resistance by regulating dormancy release [119–122]. Leveraging these associations within wild pan-genomes will yield high-density marker panels for accelerated screening and marker-assisted introgression of superior alleles into commercial scions and rootstocks.

### AI-integrated Multi-omics

With 18 successive genome assemblies and expanding pan-genome graphs now available for apple [123], providing sufficient data depth for systematic multi-omics integration. This genomic foundation has already fused disparate molecular layers into coherent regulatory maps of cold acclimation. Joint transcriptome-metabolome surveys have flagged *MdGolS5*, *MdHB7L*, and *MdHY5* as hub genes whose allelic variants correlate with raffinose, anthocyanin and ABA accumulation under freezing stress [35, 46, 124]. Integration of methylated RNA immunoprecipitation sequencing (MERIP-seq) and RNA-seq data revealed that LT-responsive m6A methylation mediated by MdMTA dynamically regulates the mRNA stability of *COR* genes [80]. The frontier of cold stress research is now being pushed by single-cell and spatial omics techniques, which resolve cell-type-specific cold responses [108, 109]. Now, graph-based pangenomes merged with transcriptome, methylome, epitranscriptome, proteome, metabolome, root microbiome, and phenome atlases enable machine-learning models to score causal alleles for cold-resistance potential, accounting for pleiotropy and developmental trade-offs, and deliver unbiased dissection of cold regulatory networks. Priority lists generated by these algorithms can then be funnelled directly into CRISPR pipelines or marker-assisted selection, accelerating the release of next-generation cold-hardy apple cultivars.

### Genome editing

CRISPR/Cas9 technology has revolutionized apple molecular breeding by enabling precise, targeted gene editing, yet its deployment for cold tolerance lags behind anthocyanin or disease-resistance traits [125–127], and has so far been successfully applied to edit genes such as *MpbZIP46* [125], *MdWRKY10* [126], and *MdMYB305* [127], *MdCNGC2* [128], and *MdDIPM4* [129]. Translating this tool into cold-resistant commercial varieties faces major hurdles. First, as an edible woody perennial, the genetic transformation efficiency of apple is unstable [130–132], making recovery of edited lines labor-intensive. Second, the long juvenile period of apple prolongs both phenotyping of cold-resistant edited offspring and subsequent breeding cycles. Therefore, further efforts should combine speed-breeding apple systems with selectable marker-free base editors delivered via ribonucleoproteins or viral vectors to accelerate the path from edited T₀ plants to field-tested, cold-resilient cultivars.

### Rootstock-mediated resilience and Grafting 

Rootstocks, the basal organ that unites scion to root system, offer the fastest deployable route to improve freezing tolerance. Cold-hardy rootstocks, including SH6, SH40, CG.3902 [133, 134], GM256 [134], Huang 6 [133], M9, B9 [134, 135], and G202 [136] confer superior resistance to the scion by lowering cell membrane permeability, enhancing SOD and POD activities, accumulating soluble sugars and proline, and increasing the ABA:IAA ratio. Grafting elite cultivars onto these rootstocks provides an immediate, non-transgenic frost-mitigating strategy while genome-edited or marker-selected scions are being generated. Future priorities should identify root-to-shoot signaling metabolites and xylem-mobile peptides that systemically prime cold defenses, offering molecular targets for next-generation rootstock design.

### Emerging bioinoculants: rhizosphere microbiome, bionanotechnology and biostimulants

Beyond genetic and grafting-based strategies, several orthogonal technologies are poised to complement apple cold-resistance toolkit. Rhizosphere microbiome regulation has emerged as a powerful modulator of cold tolerance, whereby beneficial microbial consortia enhance plant performance through modulation of soil physicochemical properties [137]. This principle is exemplified by cold-adapted bacterial consortia isolated from pea rhizosphere, which enhance rice osmotic tolerance and LT survival [138]. Concurrently, bionanotechnology represents a transformative strategy for sustainable development in mitigating plants exposure to cold stress [139]. Nano-carrier-mediated delivery of cryoprotectants maintains membrane integrity and reduces post-storage fruit weight loss without phytotoxicity [140–142]. Bioinoculants and biostimulants constitute another rapidly advancing frontier. These agents primarily promote crop growth by mobilizing soil nutrients and enhancing tolerance to abiotic stresses. Zinc-containing nanofertilizers elevate photosynthetic rate and biomass of cold-stressed maize plants while maintaining cell membrane integrity [142]. Likewise, brown-seaweed extract (BSE) enhances tomato cold stress tolerance by regulating the biosynthesis of antioxidant molecules and promoting photosynthetic capacity and fruit yield [143]. Collectively, these rapidly deployable, transgenic-free technologies can be layered onto rootstock-mediated systemic signaling and scion genetic enhancement, deepening our mechanistic insight into cold-stress responses and supporting climate-smart, sustainable intensification of apple production.
